# The Chemical Composition and Biological Properties of Coconut (*Cocos nucifera* L.) Water

**DOI:** 10.3390/molecules14125144

**Published:** 2009-12-09

**Authors:** Jean W. H. Yong, Liya Ge, Yan Fei Ng, Swee Ngin Tan

**Affiliations:** Natural Sciences and Science Education Academic Group, Nanyang Technological University, 1 Nanyang Walk, 637616 Singapore

**Keywords:** coconut water, phytohormone, auxin, cytokinin, gibberellin, inorganic ion, vitamin

## Abstract

Coconut water (coconut liquid endosperm), with its many applications, is one of the world’s most versatile natural product. This refreshing beverage is consumed worldwide as it is nutritious and beneficial for health. There is increasing scientific evidence that supports the role of coconut water in health and medicinal applications. Coconut water is traditionally used as a growth supplement in plant tissue culture/micropropagation. The wide applications of coconut water can be justified by its unique chemical composition of sugars, vitamins, minerals, amino acids and phytohormones. This review attempts to summarise and evaluate the chemical composition and biological properties of coconut water.

## 1. Introduction

**Figure 1:**
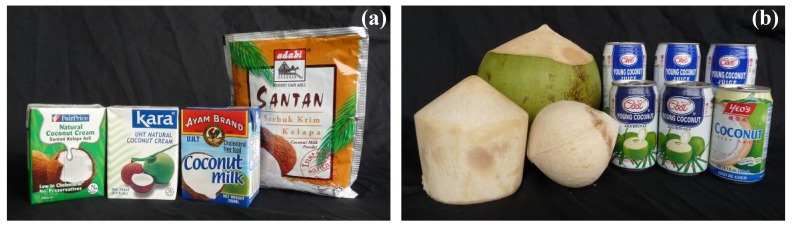
Foods and beverages made from coconut: (a) coconut milk and dried coconut milk powder; (b) canned coconut water/juice and trimmed young coconuts.

The coconut (*Cocos nucifera* L.) is an important fruit tree in the tropical regions and the fruit can be made into a variety of foods and beverages (Figure 1). The edible part of the coconut fruit (coconut meat and coconut water) is the endosperm tissue. Endosperm tissues undergo one of three main modes of development, which are the nuclear, cellular and helobial modes [1] and the development of coconut endosperm belongs to the nuclear mode. Initially, the endosperm is a liquid containing free nuclei generated by a process, in which the primary endosperm nucleus undergoes several cycles of division without cytokinesis (the process in which the cytoplasm of a single eukaryotic cell is divided to form two daughter cells). Cytokinesis then occurs, progressing from the periphery towards the center, thus forming the cellular endosperm layer. At first, the cellular endosperm is translucent and jelly-like, but it later hardens at maturity to become white flesh (coconut meat). Unlike the endosperms of other plants (e.g., wheat and corn), the cellularization process in a coconut fruit does not fill up the entire embryo sac cavity, but instead leaves the cavity solution-filled. This solution is commonly known as coconut water and it is of cytoplasmic origin [2]. Nutrients from coconut water are obtained from the seed apoplasm (surrounding cell wall) and are transported symplasmically (through plasmodemata, which is the connection between cytoplasms of adjacent cells) into the endosperm [3]. 

Coconut water should not be confused with coconut milk (Figure 1a), although some studies have used the two terms interchangeably (e.g., [4,5]). The aqueous part of the coconut endosperm is termed coconut water (Figure 1b), whereas coconut milk, also known as “santan” in Malaysia and Indonesia, and “gata” in the Philippines (Figure 1a), refers to the liquid products obtained by grating the solid endosperm, with or without addition of water [6]. Coconut water is served directly as a beverage to quench thirst (Figure 1b), while coconut milk is usually used as a food ingredient in various traditional cooking recipes (Figure 1a). The main components of coconut milk are water (*ca.* 50%), fat and protein [7], whereas coconut water contains mainly water (*ca.* 94%, Table 1). Unlike coconut water, coconut milk, which is the source of coconut oil, is generally not used in plant tissue culture medium formulations [8]. 

Compared to coconut water, there are only limited studies on the aqueous extract of coconut solid endosperm (coconut meat or copra). Mariat *et al.* used coconut meat extract in orchid tissue culture to study its effects on orchid seed germination [9]. Subsequently, Mauney *et al.* purified a growth factor from the aqueous extract of coconut meat which was found to be very potent in promoting growth of tissue cultured plants [10]. Another group, Shaw and Srivastava demonstrated the presence of purine-like substances in coconut meat extract [11]. The purine-like substances were able to delay senescence (the process of ageing in plants) in detached cereal leaves, which exhibited similar known physiological effects of cytokinins. Zakaria *et al.* showed that the aqueous extract of coconut meat exhibited anti-inflammatory and wound healing properties when tested on mice [12].

Conversely, coconut water has been extensively studied since its introduction to the scientific community in the 1940s. In its natural form, it is a refreshing and nutritious beverage which is widely consumed due to its beneficial properties to health, some of which are based on cultural/traditional beliefs [2,5,6,7,8,13,14,15]. It is also believed that coconut water could be used as an important alternative for oral rehydration and even so for intravenous hydration of patients in remote regions [13]. Coconut water may also offer protection against myocardial infarction [15]. Interestingly, a study has shown that regular consumption of either coconut water or mauby (a liquid extracted from the bark of the mauby tree, *Colubrina arborescens*), or particularly, a mixture of them, is effective in bringing about the control of hypertension [16].

Apart from that, coconut water is widely used in the plant tissue culture industry [17,18,19,20]. The extensive use of coconut water as a growth-promoting component in tissue culture medium formulation can be traced back to more than half a century ago, when Overbeek *et al.* first introduced coconut water as a new component of the nutrient medium for callus cultures in 1941 [17]. From a scientific viewpoint, the addition of coconut water to the medium is rather unsatisfactory, as it precludes the possibility for investigating the effects of individual components of the medium with any degree of accuracy. The question of which components cause the growth stimulation arose immediately. Besides its nutritional role, coconut water also appears to have growth regulatory properties, e.g., cytokinin-type activity [8]. 

Some of the most significant and useful components in coconut water are cytokinins, which are a class of phytohormones [21]. The first cytokinin, *N*^6^-furfuryladenine (kinetin) was isolated from an autoclaved sample of herring sperm DNA in 1955 [22,23]. In 1963, Letham isolated *trans*-zeatin, the first naturally-occurring cytokinin, from a plant source (unripe corn seeds) [24]. In addition to various plant-related functions, it was also found that some cytokinins (e.g., kinetin and *trans*-zeatin) showed significant anti-ageing, anti-carcinogenic, and anti-thrombotic effects [25,26].

Furthermore, micronutrients (nutrients needed in small quantities) such as inorganic ions and vitamins in coconut water play a vital role in aiding the human body antioxidant system [27]. Hypermetabolism gives rise to an increased production of reactive oxygen species (or free radicals), as a result of increased oxidative metabolism. Such increase in free radicals will cause oxidative damage to the various components of the human cell, especially the polyunsaturated fatty acids in the cell membrane, or to the nucleic acids in the nucleus [27]. Fortunately, living organisms have well developed antioxidant systems to neutralize the most detrimental effects of these oxidizing species. Micronutrients have important functions in this aspect. For example, they act directly to quench free radicals by donating electrons, or indirectly as a part of metallo enzymes (a diverse class of enzymes that require a catalytic metal ion for their biological activity) such as glutathione peroxidase (selenium) or superoxide dismutase (zinc, copper) to catalyse the removal of oxidizing species [28].

Other components found in coconut water include sugars, sugar alcohols, lipids, amino acids, nitrogenous compounds, organic acids and enzymes [20,29,30,31], and they play different functional roles in plant and human systems due to their distinct chemical properties. The myriad of compounds, both known and unknown, provide coconut water with the special biological properties that is known to the typical layman. In this paper, we will present a summary on the chemical composition of the known compounds in coconut water.

## 2. Chemical Composition of Coconut Water

### 2.1. Phytohormones

Phytohormones are a group of naturally occurring organic compounds that play crucial roles in regulating plant growth in a wide range of developmental processes. Initially, the term phytohormone was synonymous with auxin. Later on, the other plant growth regulators such as gibberellins (GAs), ethylene, cytokinins, and abscisic acid (ABA) were categorized together with auxins as the “classical five” hormones [21]. Coconut water contains auxin, various cytokinins, GAs and ABA (Table 2) [4,32,33,34,35].

#### 2.1.1. Auxin

Coconut water contains indole-3-acetic acid (IAA), the primary auxin in plants [34,35]. IAA is a weak acid (pKa = 4.75) that is synthesized in the meristematic regions located at the shoot apex and subsequently transported to the root tip in plants [36]. For many years, tryptophan was assumed to be the precursor of IAA and this was later confirmed using experiments involving seedlings of *Phaseolus vulgaris* subjected to stable isotope labeling studies [37]. IAA occurs not only in the free form, but is also conjugated to various amino acids, peptides, or carbohydrates. These IAA conjugates are biologically inactive and appear to be the IAA storage forms in seeds and are probably involved in hormonal homeostasis [38].

Auxin is implicated in many regulatory processes in plants especially those relating to plant growth and development [39,40]. Auxin functions in the relay of environmental signals such as light and gravity, the regulation of branching processes in shoots and roots, and as discovered more recently, the patterned differentiation of cells in meristems and immature organs [39]. Undoubtedly, it is a versatile spatial-temporal signal. Auxin transport generates auxin concentration maxima and gradients within tissues that are instrumental in the diverse regulation of various plant developmental processes, including embryogenesis, organogenesis, vascular tissue formation and tropisms. The unique signal-molecule transport mechanism of auxin to a large extent underlies the remarkable developmental plasticity of plants that allows their growth and architecture to fit the environment changing [41].

#### 2.1.2. Cytokinins

Cytokinins, being able to induce plant cell division, were discovered in the 1950s [22,42,43]. Natural cytokinins are *N^6^*-substituted adenine derivatives with various substituted groups, and the physicochemical behaviour of cytokinins is a function of side chain(s), sugar, phosphate and degree of purine ring and/or side chain modification [43]. The auxin-cytokinin hypothesis predicted that cytokinins, together with auxins, play an essential role in plant morphogenesis by controlling the formation of roots and shoots and moderating their relative growth [42]. Cytokinins are a class of phytohormones that exert various roles in the different aspects of plant growth and development, e.g., cell division, formation and activity of shoot meristems, induction of photosynthesis gene expression, leaf senescence, nutrient mobilization, seed germination, root growth and stress response [22,42,43,44,45,46]. Evidently, cytokinin-deficient plants generally develop stunted shoots with smaller apical meristems. The plastochrone of these cytokinin-deficient plants is prolonged, and leaf cell production is only 3-4% of wild type plants (with normal cytokinin metabolism), indicating an absolute role of cytokinins in leaf growth. Cytokinins are required during leaf formation, both to drive the cell division cycle at normal rates and to obtain the required number of divisions in order to produce a normal leaf size [42]. In addition, cytokinins are also involved in promoting the transition from undifferentiated stem cells to differentiated tissues [47]. Unlike the growth-promoting role of cytokinins in the shoot apical meristem, cytokinins have a negative regulatory function in root growth whereby it suppresses cell division in plant roots [42]. 

Furthermore, cytokinins play an important role in retarding or even reversing leaf senescence [44,45,46]. Gan and Amasino reported on the three approaches used to investigate the inhibitory role of the cytokinins in plant senescence: the external application of cytokinins, the measurement of endogenous cytokinin levels before and during senescence, and the manipulation of endogenous cytokinin production in transgenic plants. However, externally applied cytokinins were not always effective in blocking the senescence of excised leaves. The effect of cytokinins on senescence can also vary under different experimental conditions. These studies revealed an inverse correlation between cytokinin levels and the progression of senescence in a variety of tissues and plant species. Cytokinins can interfere with senescence in detached tissues of dicotyledons and monocotyledons but are often less effective in attached tissues. In addition, cytokinin levels, as well as the capacity to synthesize cytokinin, decline with the progression of leaf senescence [48].

Coconut water is an important additive in the tissue culture media of several plants, including orchids and traditional Chinese medicinal herbs. The cytokinins found in coconut water support cell division, and thus promote rapid growth. They are mostly used to propagate protocorm-like bodies of orchids in plant industries [49]. However, it should be noted that cytokinins cannot completely substitute coconut water’s effects. This is due to the presence of other phytohormones (such as auxin and gibberellins [33,34,35]) or even undefined chemical components which may exert synergistic effects with cytokinins. One advantage of coconut water is that it results in considerable plant cell proliferation without increasing the number of undesirable mutations [20]. Coconut water contains various cytokinins (Table 2). For this review, only kinetin, *trans*-zeatin and their derivatives will be discussed in greater detail as they are known to possess medicinal values [26,50,51,52].

##### *N*^6^-Furfuryladenine (Kinetin)

The first cytokinin, kinetin was discovered by Miller *et al.* in Wisconsin. It was a degradation product of herring sperm DNA and was found to be able to promote cell division in plants [22,23]. Kinetin was previously assumed to be an unnatural and synthetic compound, until in 1996 Barciszewski *et al.* detected it in freshly extracted cellular DNA from human cells and in plant cell extracts [53]. And recently, Ge *et al.* identified kinetin and kinetin riboside from coconut water [54].

Being one of the cytokinins, kinetin has the effects on the plant developmental processes that could be influenced by cytokinins, such as leaf expansion and seed germination. Most importantly, kinetin is well known for its ability to retard senescence in plants [48,55,56,57,58]. 

Recently, its strong anti-ageing effects on human skin cells and fruitflies (*Zaprionus paravittiger*) were also reported [26,59,60]. Kinetin slowed down the ageing processes, and prolonged the lifespan of fruitflies, which was mainly due to a reduction in age-specific death rates throughout the adult lifespan [59]. In addition, kinetin enhanced cell division and led to fewer cells being arrested at the G_0_/G_1_ phase, thus delaying the ageing of endothelial cells and increasing cell proliferation and metabolic capacity [61]. Most importantly, the anti-ageing effects of kinetin did not increase the cell culture lifespan in terms of maximum proliferative capacity *in vitro*, in contrast to many other anti-ageing factors which are known to promote carcinogenesis under certain conditions [26,62]. Kinetin was shown to delay the onset of several cellular and biochemical characteristics associated to cellular ageing in human skin fibroblast cultures [26]. Based on the results obtained from studies on the anti-ageing effects of kinetin on human skin cells [63], skin care products containing kinetin were subsequently developed to treat photo-damaged skin [64].

Recent research evidence revealed that oxidative DNA damage plays an important role in cancer development and that dietary antioxidants can provide effective protection against oxidative damage [65]. Kinetin was shown to act as a strong antioxidant both under *in vitro* and *in vivo* conditions. A study done by Olsen *et al.* demonstrated that kinetin protected DNA from oxidative damage mediated by the Fenton reaction. Kinetin inhibited the formation of 8-oxo-2’deoxyguanosine, which is a common marker of oxidative damage in DNA [66]. In addition, kinetin was found to inhibit oxidative and glycoxidative protein damage generated *in vitro* [67]. The anti-oxidative properties of kinetin suggested that it may also prevent the oxidative damage of unsaturated fatty acids located within the cell membranes [68].

Kinetin riboside exhibited a cytotoxic effect on plant crown-gall cells [69]. Its anti-proliferative and apoptogenic effects against human cancer cells were also well documented. Studies on the inhibition of kinetin and kinetin riboside on the growth of human fibroblasts, epithelium and mammary carcinoma were conducted [70,71,72]. A recent study revealed that kinetin riboside could induce apoptosis in HeLa and mouse melanoma B16F-10 cells [50]. Moreover, kinetin riboside also has significant effects on inhibiting the growth of human heptamoa (HepG2) cells [73]. Cabello *et al.* later found that the cytotoxic effects of kinetin riboside stemmed from its ability to induce rapid ATP depletion, creating genotoxic stress which activates p21 and other stress response genes [74]. Furthermore, a research group from the Mayo clinic (USA) identified kinetin riboside from a chemical library screen as the suppressor of cyclin D1 and D2 (CCND1 and CCND2) expression, showing that kinetin riboside could potentially act as a therapeutic agent for multiple myeloma [75].

Besides anti-ageing and anti-cancer effects, kinetin has effective anti-platelet properties, and may be a potential therapeutic agent for treating arterial thrombosis. Kinetin inhibited platelet aggregation in human platelets when stimulated by an agonist [76], and could therefore help to prevent blood clots [77,78].

##### *trans*-Zeatin

*trans*-zeatin was the first naturally-occurring cytokinin identified from a plant source (*Zea mays*) by Letham [24]. In 1974, Letham identified *trans*-zeatin in coconut water [79,80], and a year later, van Stadens and Drewes verified the presence of both *trans*-zeatin and *trans*-zeatin riboside in coconut water [81]. *trans*-zeatin riboside is the most abundant type of cytokinin found in coconut water (Table 2). *trans*-zeatin is normally used to induce plantlet regeneration from callus in plant tissue culture. Based on experimental data, *trans*-zeatin plays a key role in the G_2_-M transition of tobacco cells. It was found to override the blockade of mitosis caused by lovastatin which inhibits cytokinin biosynthesis and controls cellular entry in mitosis [82]. 

Recent studies showed that *trans*-zeatin can be a potential drug to treat neural diseases. Some researchers found that *trans*-zeatin actually possesses an inhibitory effect on acetylcholinesterase and it can be used to treat Alzheimer’s disease or related neural dysfunctions, such as dementia [51,52]. Acetylcholinesterase degrades the neural compounds that mediate neural transmission, and thus by blocking its action, synaptic transmission can be improved. Another recent study also found that *trans*-zeatin can prevent amyloid *β*-protein formation, which has a causal role in the development and progress of Alzheimer’s disease [83]. On the other hand, like kinetin, *trans*-zeatin also exhibited anti-ageing effects on human fibroblast cells [84].

#### 2.1.3. Gibberellins (GAs)

GAs are a class of phytohormones which exert certain effects on plant growth and development, in aspects such as seed germination, epidermal cell elongation, leaf expansion and flower development. The main biological action of GAs is their ability to stimulate the elongation of plant shoots and induce the growth of stems in rosette and dwarfish forms. Together with auxins, GAs stimulate cambial activity and in effect, causing the formation of large xylem and phloem cells in woody plants [85,86,87]. Apart from the vital roles played in plants, recent study also showed that gibberellin derivatives have anti-tumor bioactivities [88]. Chemically, all known GAs are gibberellic acids (a family of diterpenoids acids), and there are currently 136 members of GAs identified based on their chemical structures. GAs are numbered neither by their structural information nor by their functions, but rather in the order of their identification [85,87]. GA_1_ and GA_3_ were successfully detected and quantified in coconut water [33,89].

### 2.2. Inorganic ions

Inorganic ions are required for normal cellular function, and are critical for enzyme activation, bone formation, hemoglobin function, gene expression, and the metabolism of amino acids, lipids and carbohydrates [90,91,92,93]. Coconut water contains a variety of inorganic ions (Table 1) [20,29,30,31] and these ions contribute to the therapeutic value inherent in coconut water. As the basic ion composition of coconut water can replenish the electrolytes of the human body excreted through sweat, such as sodium, potassium, magnesium and calcium, it can serve as an effective rehydration drink [94]. The concentration of these electrolytes in coconut water generates an osmotic pressure similar to that observed in blood [95], and it also does not affect hemostasis (plasma coagulation) [14]. As a result, coconut water can be used as a short term intravenous hydration fluid under certain emergency situations [13]. Interestingly, Anurag and Rajamohan showed that coconut water has cardioprotective effects in experimental myocardial infarction induced in rats and this was probably attributed to the rich content of mineral ions in coconut water, especially potassium [15]. 

### 2.3. Vitamins 

Vitamins, which are essential for the normal functioning of the human body, are also found in coconut water. Greater consumption of fruits and vegetables is associated with the reduced risk of cardiovascular disease, stroke, and cancers of the mouth, pharynx, esophagus, lungs, stomach, and colon [96,97,98,99], because they contain vitamins and minerals vital for normal physiological functions [86]. Coconut water contains vitamins B_1_, B_2_, B_3_, B_5_, B_6_, B_7_ and B_9_ (Table 1). The B vitamins are water-soluble and are required as coenzymes for enzymatic reactions essential for cellular function [100]. 

Vitamin B_6_ (which includes pyridoxal, pyridoxine and pyridoxamine) serves as a coenzyme in various enzymatic reactions, such as the transamination and decarboxylation reactions [101]. For example, it is the coenzyme of γ-cystathionase [102], which catalyses the cleavage of cystathionine, releasing α-ketobutyrate and cystein [103]. The α-ketobutyrate molecule is subsequently converted into succinyl-CoA and fed to the tricarboxylic acid (TCA) cycle while cystein is involved in protein and gluthathione biosynthesis [104,105]. Vitamin B_6_ deficiency can affect various processes of the body, such as inflammation and renal function [100]. 

Coconut water contains folate (Table 1), also known as vitamin B_9_. It was identified in the late 1930’s as the nutrient required to reduce anemia during pregnancy [106]. It can prevent mitochondrial toxicity induced by methanol metabolites. In addition, the active form of folate, 5-methyltetrahydro-folate is believed to be one of the central methyl donors required for mitochondrial protein and nucleic acid synthesis [28]. Lower blood levels of vitamin B_6_ and folate can increase the risk for atherosclerosis and other vascular diseases [107]. Another study found that high plasma levels of vitamin B_6_ and folate may reduce the risk for breast cancer [108]. In addition to vitamin B, coconut water also contains vitamin C (total ascorbic acid, Table 1), which is an important dietary antioxidant [28,63]. 

## 3. Conclusions

Coconut water, being a refreshing beverage, provides important health benefits. The chemical components which contribute to its bioactivity are essential to the plant industry, biotechnology and biomedical fields. Undoubtedly, cytokinins are currently the most important components in coconut water. Significant advances were made in understanding the biological functions of the various cytokinins in both plant and human systems. The potential anti-cancer properties of specific cytokinins could bring encouraging and novel perspectives in finding cures for the different types of cancers. The recent discovery of other medicinal values of coconut water signifies a good potential in improving human health. Better insights and understanding of the functions and properties of the individual components of coconut water will, therefore, help us to better utilise this marvellous and multi-dimensional liquid with special biological properties from nature.

## 4. Future Studies

The chemical composition of coconut water is affected by several factors. Jackson *et al.* showed that coconut water of different coconut varieties contains different concentration of compounds, and that the chemical contents also varied during the different stages of maturity [109]. Soil and environmental conditions also affect the chemical profile of coconut water. A study which was done in Brazil demonstrated that the physical properties of coconut water were affected by varying nitrogen and potassium application [110]. Hence, future studies should be carried out to determine the factors that produce the desirable chemical composition for a specific purpose. Breeding studies can also be carried out to produce coconut water enriched with specific chemical compounds.

Although coconut water is already well studied in terms of its chemical content, there may still be unknown solutes which contribute to its special biological effects. With the development of more advanced detection techniques, screening can be intensified to detect novel compounds of medicinal values present in coconut water.

## Figures and Tables

**Table 1:** Chemical composition of coconut water [20,29,30,31].

**Source information**	**[20]**	**[31]**	**[29]**				**[30]**	
Coconut type		**young**	**young green**	**mature green**	**mature**	**mature (autoclaved)**	**young**	**mature**
Average Weight of Coconut (g)		206 (water)					565	393
Age of coconut							6 months	12 months
Source of coconut			Deerfield Beach, FL		Dominican Republic			
**Proximates**		*(g/100g)*					*(g/100g )*	
Water		94.99					94.18	94.45
Dry		5.01					5.82	5.55
Energy value		19 kcal (79 kJ)						
Protein		0.72					0.12	0.52
Total lipid (fat)		0.2					0.07	0.15
Ash		0.39					0.87	0.47
Carbohydrate, by difference		3.71					4.76	4.41
Fiber, total dietary		1.1					ND*	ND*
**Sugars**	*(mg/mL)*	*(g/100g)*	*(mg/mL )*				*(g/100g )*	
Total		2.61	9.16	21.68	13.87	15.20	5.23	3.42
Sucrose	9.18		0.93	9.18	8.90	10.70	0.06	0.51
Glucose	7.25		3.93	7.25	2.46	2.02	2.61	1.48
Fructose	5.25		4.30	5.25	2.51	2.48	2.55	1.43
**Sugar alcohols**	Present ^a^			*(mg/L )*				
Mannitol	0.8			0.80				
Sorbitol	15 ^d^			15.00				
Myo-inositol	0.01			0.01				
Scyllo-inositol	0.05			0.05				
**Inorganic ions**	*(mg/100g)*	*(mg/100g)*		*(mg/100g )*			*(mg/100g )*	
Calcium, Ca		24					27.35	31.64
Iron, Fe	0.01	0.29		0.01			0.02	0.02
Magnesium, Mg	30	25		30			6.40	9.44
Phosphorus, P	37	20		37			4.66	12.77
Potassium, K	312	250		312			203.70	257.52
Sodium, Na	105	105		105			1.75	16.10
Zinc, Zn		0.1					0.07	0.02
Copper, Cu	0.04	0.04		0.04			0.01	0.03
Manganese, Mn		0.142					0.12	0.08
Selenium, Se		0.001						
Chlorine, Cl	183			183				
Sulfur, S	24			24			0.58	
Aluminium, Al							0.07	0.06
Boron, B							0.05	0.08
**Vitamins**	*(mg/mL)*	*(mg/100g )*		*(mg /L )*			*(mg /100 dm ^3^)*	
Vitamin C, total ascorbic acid		2.4					7.41	7.08
Thiamin (B1)		0.03		Trace			Trace	0.01
Riboflavin (B2)		0.057		0.01			0.01	0.01
Niacin (B3)		0.08		0.64			ND*	ND*
Pantothenic acid (B5)	0.52	0.043		0.52				
Pyridoxine (B6)		0.032		Trace			ND*	ND*
Folate, total		0.03						
Folic acid	0.003	0		0.003				
Folate, food		0.003						
Folate, Dietary Folate Equivalent (DFE)		3 (µg_DFE)						
Biotin	0.02			0.02				
Nicotinic acid (Niacin)	0.64			0.64				
**Lipids**		*(g/100g )*					*(g/100g )*	
Total		0.2					0.0733	0.1482
**Fatty acids, total saturated**		0.176					0.03	0.1
6:00		0.001						
8:00		0.014					ND*	ND*
10:00		0.011					0.0007	0.0028
12:00		0.088					0.002	0.0274
14:00		0.035					0.0023	0.019
16:00		0.017					0.0219	0.032
17:00							0.0009	0.0016
18:00		0.01					0.0039	0.0108
20:00							0.0016	0.0033
**Fatty acids, total monounsaturated**		0.008					0.03	0.02
16:1 undifferentiated		0					0.0011	0.0007
18:1 undifferentiated		0.008					0.0194	0.015
20:1 undifferentiated							0.0049	0.0019
22:1 undifferentiated							0.0011	0.0023
**Fatty acids, total polyunsaturated**		0.002					0.0128	0.0054
18:2 *n-6*undifferentiated		0.002					0.0114	0.0032
20:4 *n-6*							0.0014	0.0022
**Amino acids**	*(µg/mL)*	*(g/100g )*	*(µg/mL)*				*(mg/g defatted sample)*	
Alanine	312	0.037	16.40	127.30	177.10	198.00	1.13	3.88
β-Alanine	12							
γ-Aminobutyric acid	820		1.90	34.60	168.80	173.20		
Arginine	133	0.118	14.70	25.60	16.80	20.70	0.13	0.81
Asparagine and glutamine	*ca*. 60							
Aspartic acid	65	0.07	11.30	35.90	5.40	11.40	1.60	0.76
Asparagine			17.10	10.10	10.40	25.30		
Cystine	0.97-1.17 ^b^	0.014					0.00	0.00
Glutamic acid	240	0.165	9.40	70.80	78.70	104.90	3.44	3.75
Glutamine			80.00	45.40	13.40	2.00		
Glycine	13.9	0.034	1.30	9.70	13.90	18.00	0.43	0.11
Homoserine	5.2		ND*	ND*	5.20	8.80		
Histidine	Trace ^a^	0.017	3.50	6.30	Trace ^a^	Trace ^a^	0.39	0.67
Isoleucine	18	0.028					0.26	0.27
Leucine	22	0.053	6.20	37.30	31.70	33.00	0.66	0.58
Lysine	150	0.032	4.40	21.40	22.50	13.00	4.72	3.41
Methionine	8	0.013	3.50	16.90	Trace ^a^	Trace ^a^	0.22	0.21
Ornithine	22							
Phenylalanine	12	0.037	ND*	ND*	10.20	Trace ^a^	0.26	0.00
Pipecolic acid		Trace ^a^						
Proline	97	0.03	4.10	31.90	21.60	12.90	0.52	0.95
Serine	111	0.037	7.30	45.30	65.80	85.00	0.64	1.06
Tyrosine	16	0.022	0.90	6.40	3.10	Trace ^a^	0.00	0.00
Tryptophan	39	0.008					0.00	0.00
Threonine	44	0.026	2.90	16.20	26.30	27.40	0.20	0.33
Valine	27	0.044	5.60	20.60	15.10	15.50	0.91	0.82
Dihydroxyphenylaline	Present ^a^							
Hydroxyproline	Trace ^a^		Trace ^a^	4.10	Trace ^a^	8.20		
Pipecolic acid	Present ^a^	Trace ^a^						
**Nitrogeneous compounds**	*µmol/mL*							
Ethanolamine	0.01							
Ammonia	Present ^a^							
**Organic acids**	*(meq/mL)*		*(meq/mL )*				*(mg /100 DM)*	
Tartaric							1.6	2.4
Malic	34.31		9.36	34.31	11.98	14.08	317	307
Citric	0.37			0.37	0.31	0.38	ND*	24.8
Acetic							ND*	1.3
Pyridoline	0.39 mg/mL		0.43	0.39	0.18	0.27		
Succinic					0.28	0.18		
Shikimic and quinic acids, etc.	0.57							
**Enzymes**								
Acid phosphatase	Present ^a^							
Catalase	Present ^a^							
Dehydrogenase	Present ^a^							
Diastase	Present ^a^							
Peroxidase	Present ^a^							
RNA polymerases	Present ^a^							
**Phytohormones**	*(mg/mL)*			*(mg/L )*				
Auxin	0.07			0.07				
1,3- Diphenylurea				5.8				
Cytokinin	Present ^a^							
**Miscellaneous**								
Leucoanthocyanin	Present ^a^							
Phyllococosine	Present ^a^							
**Chemical properties**								
pH			4.6 to 5.6				4.7±0.1	5.2±0.1

* ND = Non detectable; ^a^ No units given; ^b^ Units: g/100g dried protein; ^d^ Units: mg/mL; ^e^ Due to contamination.

**Table 2:** Naturally-occurring phytohormones unequivocally identified in coconut water.

Source information	[4]	[32,33,34]	[35]
Coconut type		young green	mature*
**Auxin**		nM	μg mL^-1^
indole-3-acetic acid		150.6	0.25 ± 0.03
			0.75 ± 0.04
			1.46 ± 0.13
			0.71 ± 0.12
			0.78 ± 0.10
**Cytokinins**			
*N*^6^-isopentenyladenine		0.26	
dihydrozeatin		0.14	
*trans*-zeatin		0.09	
kinetin		0.31	
*ortho*-topolin		3.29	
dihydrozeatin *O*-glucoside		46.6	
*trans*-zeatin *O*-glucoside		48.7	
*trans-*zeatin riboside		76.2	
kinetin riboside		0.33	
*trans*-zeatin riboside-5’-monophosphate		10.2	
14-*O*-(3-*O*-[*β*-D-galacto-pyranosyl-(1→2) -*α*-D-galactopyranosyl- (1→3) -*α*-L- arabinofuranosyl]-4-O-(*α*-L-arabinofuranosyl)- *β*-D-galactopyranosyl)-*trans*-zeatin riboside	*Present*		
**Gibberellins**			
gibberellin 1		16.7	
gibberellin 3		37.8	
**Auxin**			
indole-3-acetic acid		150.6	
**Abscisic acid**		65.5	0.010 ± 0.002
			ND
			0.023 ± 0.002
			0.061 ± 0.019
			0.071 ± 0.018
**Salicylic acid**			1.01 ± 0.10
			0.67 ± 0.04
			1.03 ± 0.12
			1.79 ± 0.21
			1.22 ± 0.07

* Five coconut water samples were analysed.
